# Supplementary material for: Nelfinavir Inhibits the TCF11/Nrf1-Mediated Proteasome Recovery Pathway in Multiple Myeloma
Source: Cancers (Basel). 2020 Apr 25;12(5):1065. doi: 10.3390/cancers12051065 (PMC7281108; doi:10.3390/cancers12051065)
Supplement: Supplementary file 1 [file cancers-12-01065-s001.pdf]

Supplementary Materials: Nelfinavir Inhibits the TCF11/Nrf1-Mediated Proteasome Recovery Pathway in Multiple Myeloma

Dominika Fassmannová, František Sedlák, Jindřich Sedláček, Ivan Špička and Klára Grantz Šašková

Supplementary Figures

Nelfinavir inhibits TCF11/Nrf1 proteolytic processing. HEK293 cells and OPM-2 and RPMI8226 MM cells were treated with 10 µM or 20 µM nelfinavir alone or in combination with 200 nM BTZ (HEK293 cells) or 20 nM BTZ (OPM-2, RMPI8226 cells) for 16 h. Cells were lysed and subjected to immunoblot analysis visualizing TCF11/Nrf1, DDI2 and tubulin as a loading control for HEK293 cells. Ponceau staining was used as a loading control for myeloma cells. Total Nrf1 expression was quantified from the integrated fluorescence of corresponding TCF11/Nrf1 bands normalized to tubulin or the Ponceau loading control. The processed (activated) form of Nrf1 was quantified as shown as [%]. Data from 6 (HEK293 cells) and 3 (OPM-2, RPMI8226 cells) independent experiments are shown. Immunoblots are shown below.

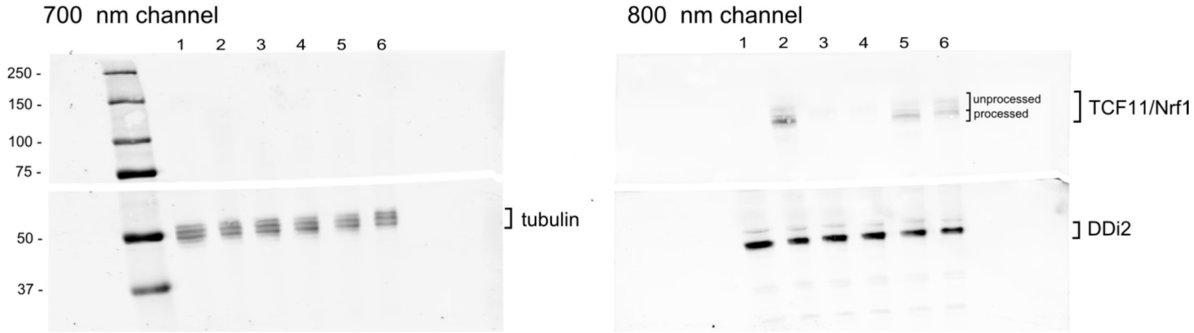

lane	Sample	Integrated Fluorescence [Raw]				Tubulin Coef.	Sum	Processed [%]
		Tubulin	Unprocessed	Processed	Sum	[sig./max.sig.]	[Corrected]	
1	DMSO	15680	-	-	-	1.00	-	-
2	BTZ	10330	4041	6855	10897	0.66	16541	63
3	NFV 10 M	12956	354	57	411	0.83	498	14
4	NFV 20 M	12032	284	8	292	0.77	381	3
5	BTZ + NFV 10 M	10232	1585	3246	4831	0.65	7403	67
6	BTZ + NFV 20 M	10479	1765	2107	3871	0.67	5793	54

Figure S1. HEK293 cells were treated with 10 µM or 20 µM nelfinavir alone or in combination with 200 nM BTZ for 16 h. Cells were lysed and subjected to immunoblot analysis visualizing TCF11/Nrf1, DDI2 and tubulin as a loading control. First independent experiment.

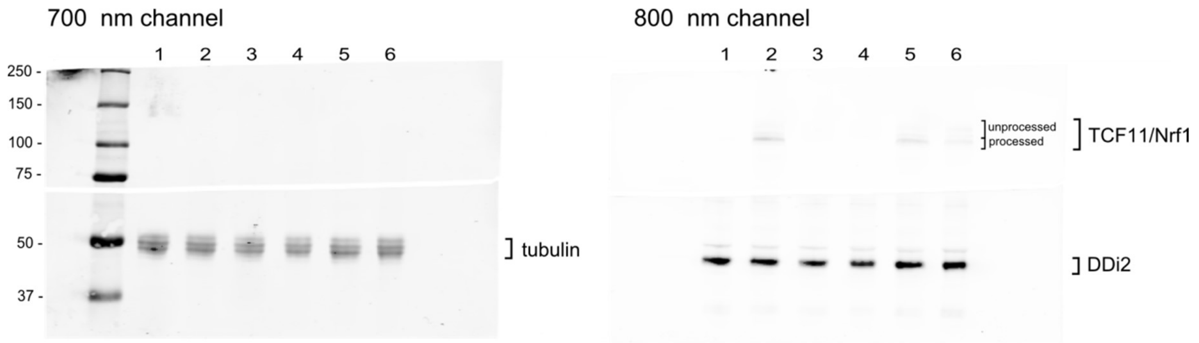

Lane	Sample	Integrated Fluorescence [Raw]				Tubulin Coef.	Sum	Processed [%]
		Tubulin	Unprocessed	Processed	Sum	[sig./max.sig.]	[Corrected]	
1	DMSO	15356	77	69	145	1.00	145	47
2	BTZ	13369	2983	5427	8410	0.87	9661	65
3	NFV 10 M	11061	205	64	268	0.72	373	24
4	NFV 20 M	9475	187	-	187	0.62	304	-
5	BTZ + NFV 10 M	12658	2715	3883	6599	0.82	8005	59
6	BTZ + NFV 20 M	12392	2248	1811	4059	0.81	5030	45

Figure S2. HEK293 cells were treated with 10 µM or 20 µM nelfinavir alone or in combination with 200 nM BTZ for 16 h. Cells were lysed and subjected to immunoblot analysis visualizing TCF11/Nrf1, DDI2 and tubulin as a loading control. Second independent experiment.

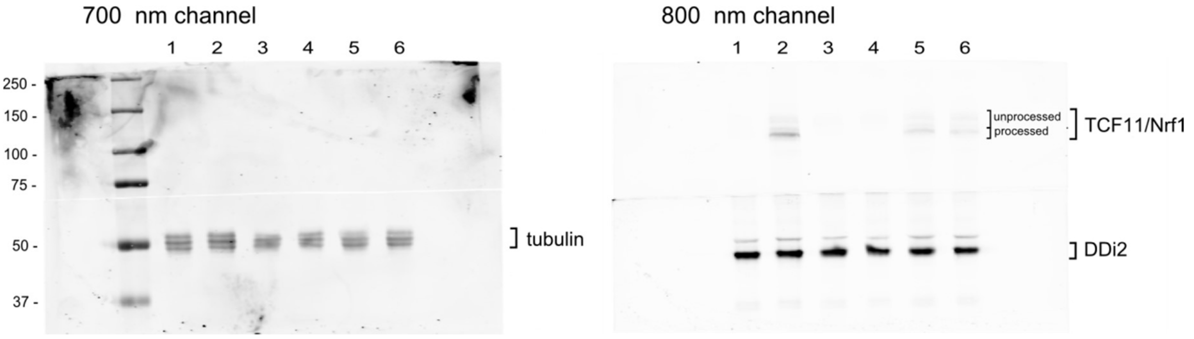

Lane	Sample	Integrated Fluorescence [Raw]				Tubulin Coef.	Sum	Processed [%]
		Tubulin	Unprocessed	Processed	Sum	[sig./max.sig.]	[Corrected]	
1	DMSO	12447	136	101	237	0.88	268	43
2	BTZ	14111	3072	6315	9387	1.00	9387	67
3	NFV 10 M	9404	450	105	555	0.67	833	19
4	NFV 20 M	8931	434	45	479	0.63	756	9
5	BTZ + NFV 10 M	10422	1837	2203	4040	0.74	5471	55
6	BTZ + NFV 20 M	10379	1594	1433	3027	0.74	4115	47

Figure S3. HEK293 cells were treated with 10 µM or 20 µM nelfinavir alone or in combination with 200 nM BTZ for 16 h. Cells were lysed and subjected to immunoblot analysis visualizing TCF11/Nrf1, DDI2 and tubulin as a loading control. Third independent experiment.

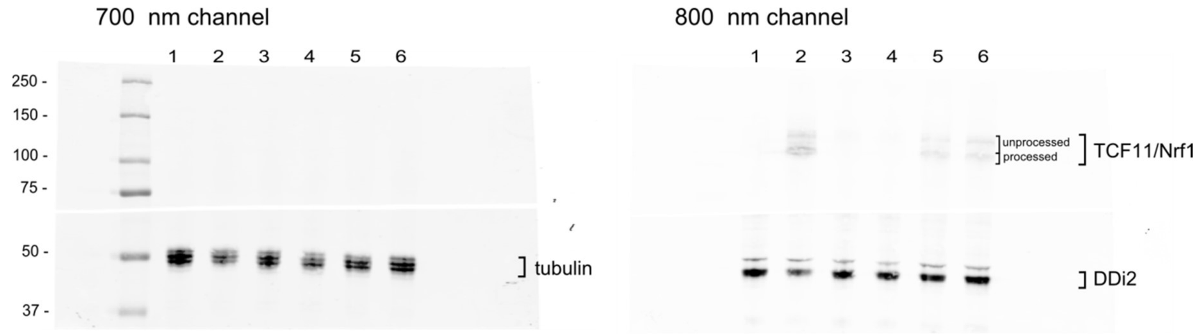

Lane	Sample	Integrated Fluorescence [Raw]				Tubulin Coef.	Sum	Processed [%]
		Tubulin	Unprocessed	Processed	Sum	[sig./max.sig.]	[Corrected]	
1	DMSO	12542	462	689	1152	1.00	1152	60
2	BTZ	6308	8064	9793	17857	0.50	35504	55
3	NFV 10 M	8278	1093	250	1343	0.66	2034	19
4	NFV 20 M	6092	624	90	714	0.49	1471	13
5	BTZ + NFV 10 M	8372	3806	3990	7796	0.67	11680	51
6	BTZ + NFV 20 M	10572	5616	4387	10004	0.84	11867	44

Figure S4. HEK293 cells were treated with 10 μM or 20 μM nelfinavir alone or in combination with 200 nM BTZ for 16 h. Cells were lysed and subjected to immunoblot analysis visualizing TCF11/Nrf1, DDI2 and tubulin as a loading control. Fourth independent experiment.

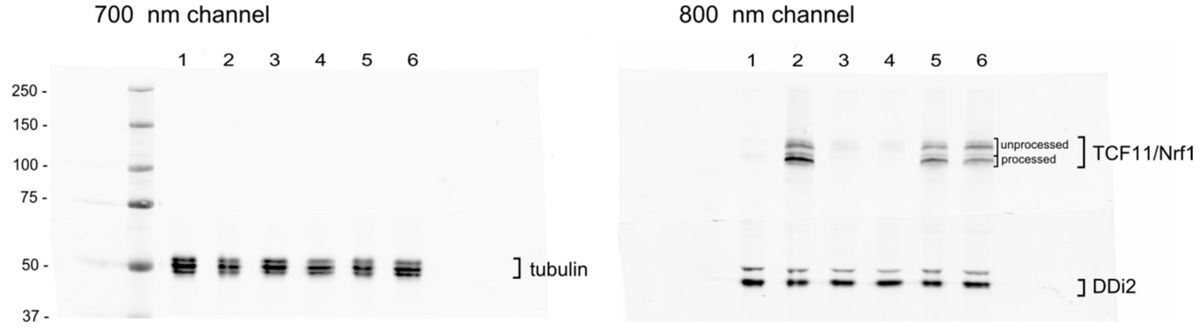

Lane	Sample	Integrated Fluorescence [Raw]				Tubulin Coef.	Sum	Processed [%]
		Tubulin	Unprocessed	Processed	Sum	[sig./max.sig.]	[Corrected]	
1	DMSO	13617	327	193	521	1.00	521	37
2	BTZ	8777	5601	7123	12724	0.64	19741	56
3	NFV 10 M	11590	739	261	1000	0.85	1175	26
4	NFV 20 M	9611	606	99	705	0.71	999	14
5	BTZ + NFV 10 M	8587	3157	3605	6761	0.63	10722	53
6	BTZ + NFV 20 M	11986	4244	2993	7237	0.88	8221	41

Figure S5. HEK293 cells were treated with 10 μM or 20 μM nelfinavir alone or in combination with 200 nM BTZ for 16 h. Cells were lysed and subjected to immunoblot analysis visualizing TCF11/Nrf1, DDI2 and tubulin as a loading control. Fifth independent experiment.

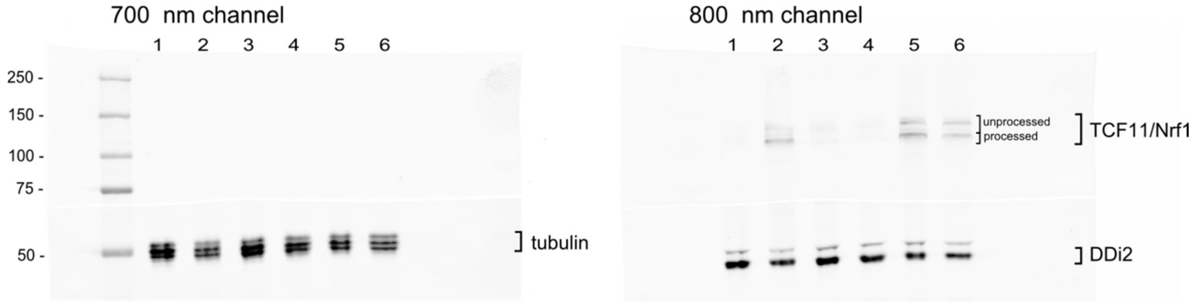

Lane	Sample	Integrated Fluorescence [Raw]				Tubulin Coef.	Sum	Processed [%]
		Tubulin	Unprocessed	Processed	Sum	[sig./max.sig.]	[Corrected]	
1	DMSO	13241	436	589	1025	1.00	1025	57
2	BTZ	9119	3332	5704	9036	0.69	13120	63
3	NFV 10 M	12958	1796	474	2270	0.98	2320	21
4	NFV 20 M	10666	1129	344	1474	0.81	1829	23
5	BTZ + NFV 10 M	8915	6461	7635	14096	0.67	20936	54
6	BTZ + NFV 20 M	9344	4238	3736	7975	0.71	11300	47

Figure S6. HEK293 cells were treated with 10 µM or 20 µM nelfinavir alone or in combination with 200 nM BTZ for 16 h. Cells were lysed and subjected to immunoblot analysis visualizing TCF11/Nrf1, DDI2 and tubulin as a loading control. Sixth independent experiment.

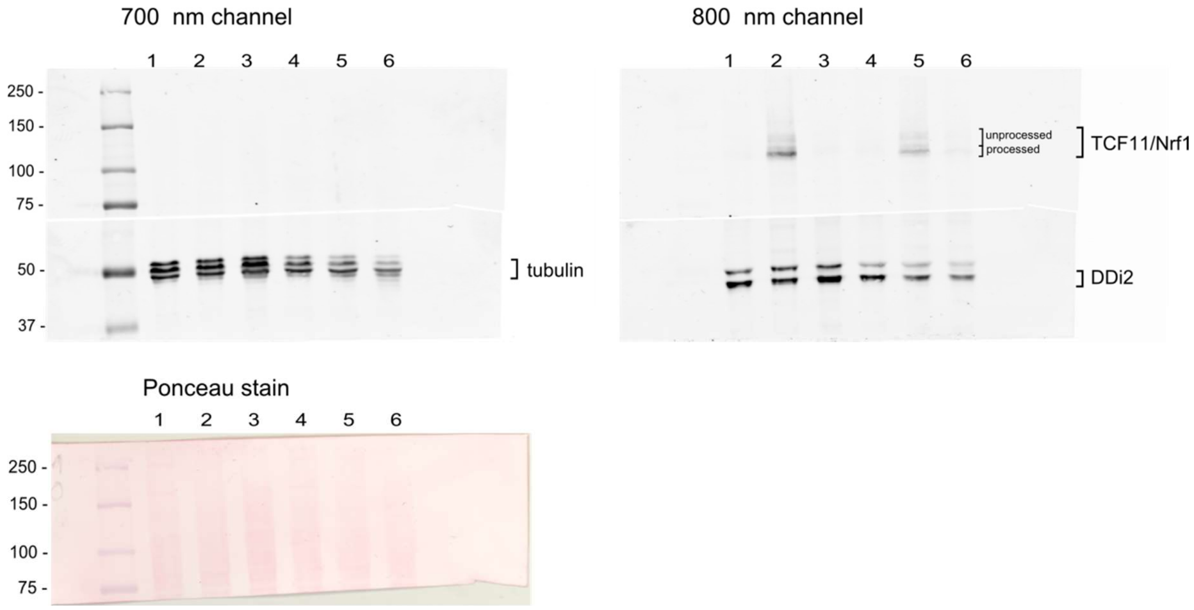

Lane	Sample	Integrated Signal	Integrated Fluorescence [Raw]			Ponceau Coef.	Sum	Processed
		Ponceau	Unprocessed	Processed	Sum	[sig./max.sig.]	[Corrected]	[%]
1	DMSO	15776	143	329	472	0.78	606	70
2	BTZ	16192	6521	8374	14894	0.80	18631	56
3	NFV 10 M	20255	290	212	502	1.00	502	42
4	NFV 20 M	15946	307	220	527	0.79	669	42
5	BTZ + NFV 10 M	14653	3304	3784	7088	0.72	9797	53
6	BTZ + NFV 20 M	12221	541	538	1079	0.60	1788	50

Figure S7. OPM-2 cells were treated with 10 µM or 20 µM nelfinavir alone or in combination with 20 nM BTZ for 16 h. Cells were lysed and subjected to immunoblot analysis visualizing TCF11/Nrf1 and DDI2. Ponceau staining was used as a loading control. First independent experiment.

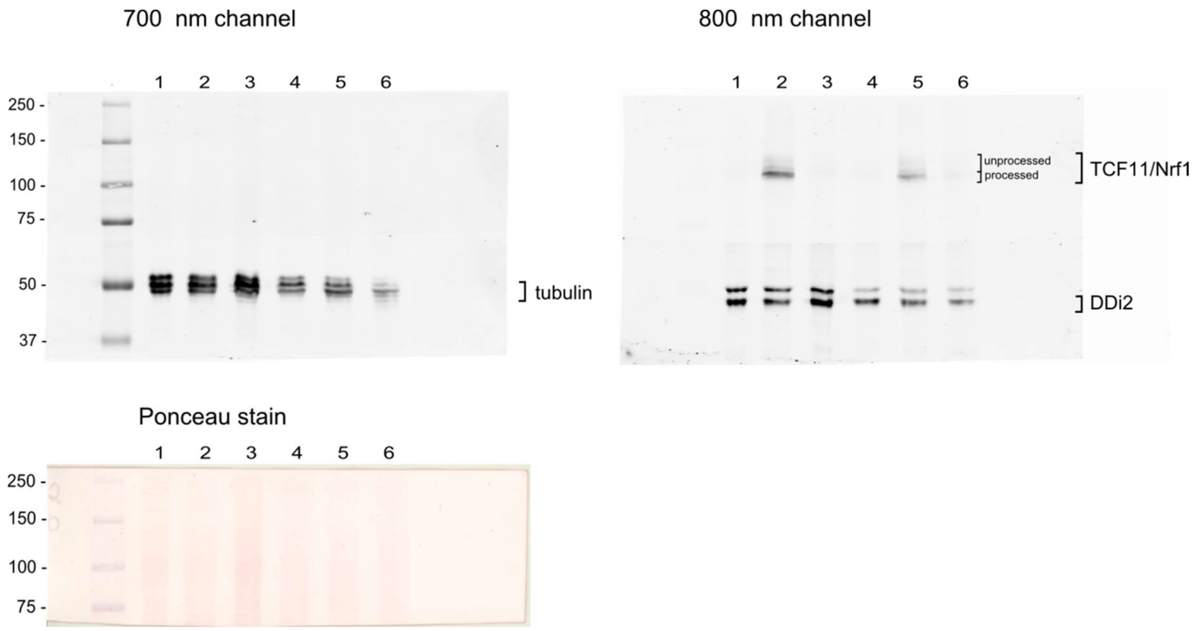

Lane	Sample	Integrated Signal Ponceau	Integrated Fluorescence [raw] Unprocessed	Processed	Sum	Ponceau Coef. [sig./max.sig.]	Sum [Corrected]	Processed [%]
1	DMSO	12065	314	361	675	0.57	1194	53
2	BTZ	10909	3410	8805	12215	0.51	23896	72
3	NFV 10 M	21342	252	233	485	1.00	485	48
4	NFV 20 M	9907	395	305	701	0.46	1509	44
5	BTZ + NFV 10 M	9566	2234	3822	6057	0.45	13512	63
6	BTZ + NFV 20 M	10792	372	419	791	0.51	1564	53

Figure S8. OPM-2 cells were treated with 10 µM or 20 µM nelfinavir alone or in combination with 20 nM BTZ for 16 h. Cells were lysed and subjected to immunoblot analysis visualizing TCF11/Nrf1 and DDI2. Ponceau staining was used as a loading control. Second independent experiment.

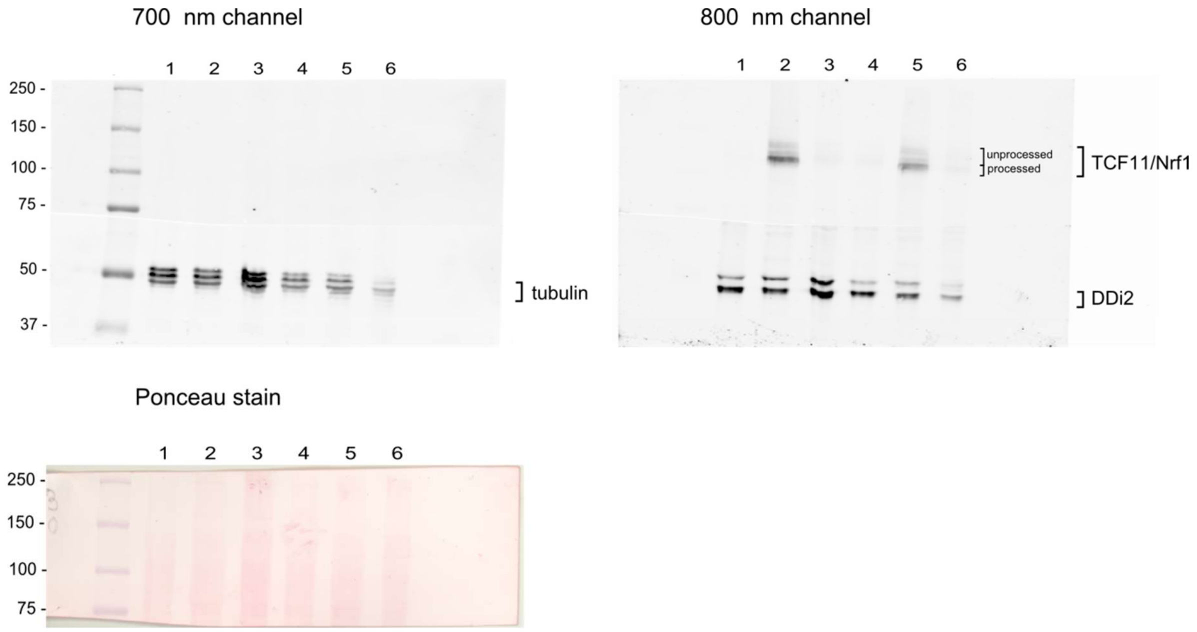

Lane	Sample	Integrated Signal	Integrated Fluorescence [Raw]			Ponceau Coef.	Sum	Processed
		Ponceau	Unprocessed	Processed	Sum	[sig./max.sig.]	[Corrected]	[%]
1	DMSO	11043	112	177	289	0.41	703	61
2	BTZ	16175	4290	9948	14238	0.60	23641	70
3	NFV 10 M	26859	378	585	963	1.00	963	61
4	NFV 20 M	17421	249	461	710	0.65	1095	65
5	BTZ + NFV 10 M	17053	3023	5774	8796	0.63	13855	66
6	BTZ + NFV 20 M	14695	588	519	1107	0.55	2023	47

Figure S9. OPM-2 cells were treated with 10 μM or 20 μM nelfinavir alone or in combination with 20 nM BTZ for 16 h. Cells were lysed and subjected to immunoblot analysis visualizing TCF11/Nrf1 and DDI2. Ponceau staining was used as a loading control. Third independent experiment.

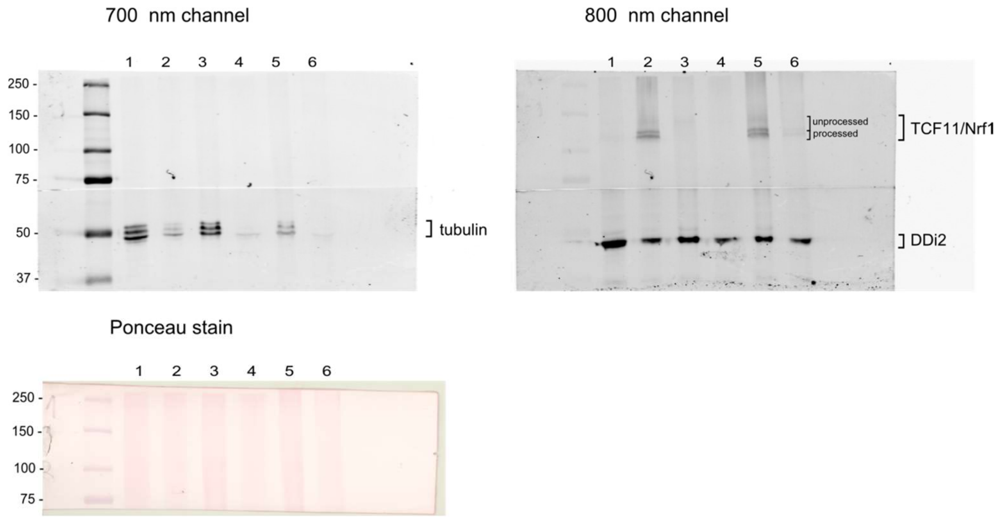

Lane	Sample	Integrated Signal	Integrated Fluorescence [Raw]			Ponceau Coef.	Sum	Processed
		Ponceau	Unprocessed	Processed	Sum	[sig./max.sig.]	[Corrected]	[%]
1	DMSO	30753	350	448	798	1.00	801	56
2	BTZ	21687	5055	3998	9053	0.70	12885	44
3	NFV 10 M	30866	883	237	1120	1.00	1120	21
4	NFV 20 M	20057	-	-	-	0.65	-	-
5	BTZ + NFV 10 M	29628	5443	3647	9090	0.96	9470	40
6	BTZ + NFV 20 M	18990	1180	733	1913	0.62	3110	38

Figure S10. RPMI8226 cells were treated with 10 μM or 20 μM nelfinavir alone or in combination with 20 nM BTZ for 16 h. Cells were lysed and subjected to immunoblot analysis visualizing TCF11/Nrf1 and DDI2. Ponceau staining was used as a loading control. First independent experiment.

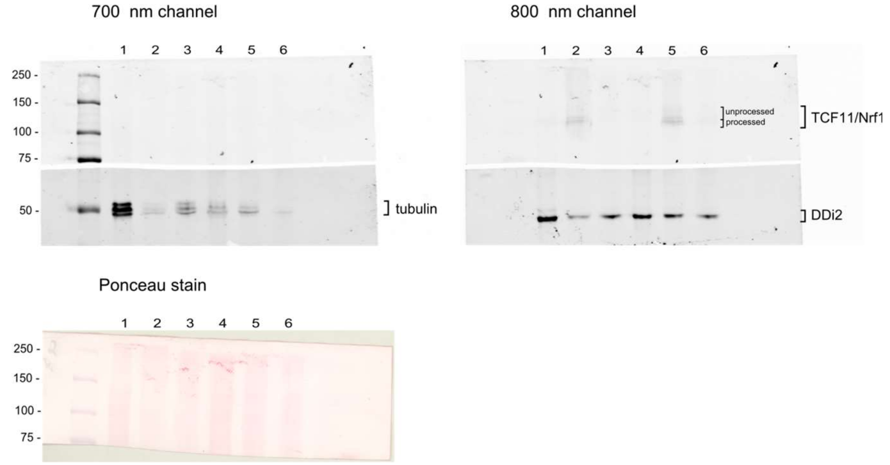

Lane	Sample	Integrated Signal	Integrated Fluorescence [Raw]			Ponceau Coef.	Sum	Processed
		Ponceau	Unprocessed	processed	Sum	[sig./max.sig.]	[Corrected]	[%]
1	DMSO	27343	998	1018	2016	0.65	**3096**	**51**
2	BTZ	22845	3278	4587	7865	0.54	**14456**	**58**
3	NFV 10 M	28043	680	110	790	0.67	**1183**	**14**
4	NFV 20 M	41985	1060	188	1248	1.00	**1248**	**15**
5	BTZ + NFV 10 M	31528	6689	6514	13203	0.75	**17582**	**49**
6	BTZ + NFV 20 M	23376	1542	1130	2672	0.56	**4798**	**42**

Figure S11. RPMI8226 cells were treated with 10 μM or 20 μM nelfinavir alone or in combination with 20 nM BTZ for 16 h. Cells were lysed and subjected to immunoblot analysis visualizing TCF11/Nrf1 and DDI2. Ponceau staining was used as a loading control. Second independent experiment.

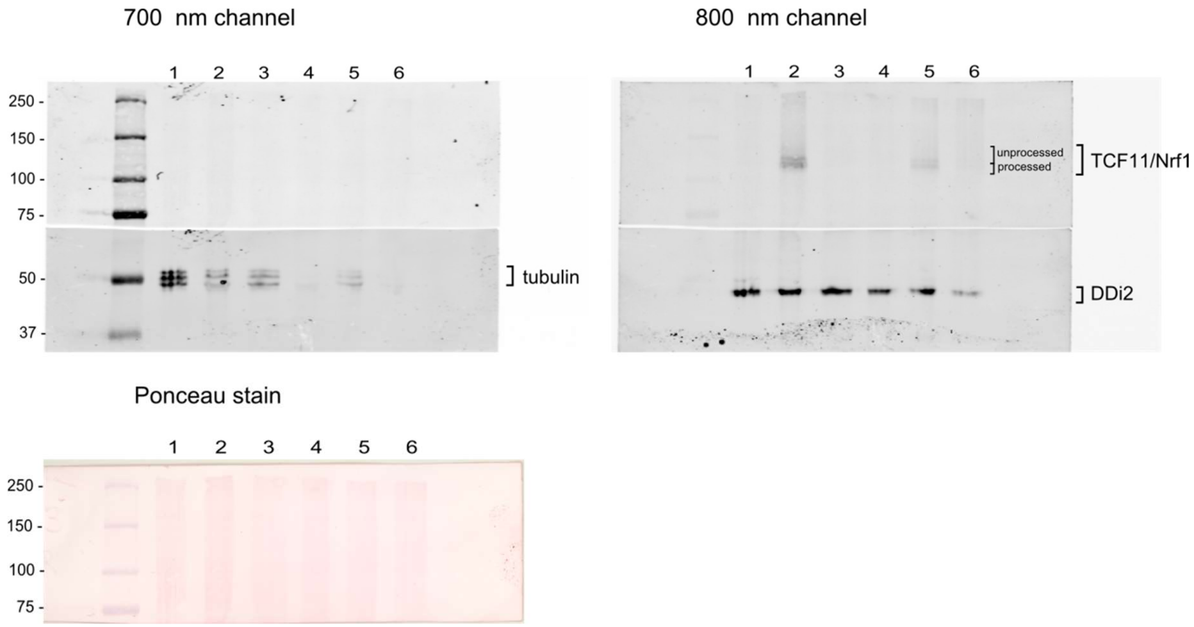

Lane	Sample	Integrated Signal Ponceau	Integrated Fluorescence [Raw] Unprocessed	Processed	Sum	Ponceau Coef. [sig./max.sig.]	Sum [Corrected]	Processed [%]
1	DMSO	25270	-	-	764	0.93	824	-
2	BTZ	27245	7706	7568	15274	1.00	15274	50
3	NFV 10 M	16611	-	-	662	0.61	1086	-
4	NFV 20 M	17584	-	-	322	0.65	500	-
5	BTZ + NFV 10 M	19316	2412	2426	4838	0.71	6825	50
6	BTZ + NFV 20 M	21089	1021	368	1389	0.77	1794	26

Figure S12. RPMI8226 cells were treated with 10 µM or 20 µM nelfinavir alone or in combination with 20 nM BTZ for 16 h. Cells were lysed and subjected to immunoblot analysis visualizing TCF11/Nrf1 and DDI2. Ponceau staining was used as a loading control. Third independent experiment.

Does NFV Activates Nrf2 Pathway and Does It Have an Effect on Proteasome Re-Synthesis When Nrf1 Pathway Is Impaired?

To evaluate our working hypothesis that NFV, by activation of the ER and oxidative stress, activates also Nrf2 pathway, we performed RT-qPCR analysis of HEK293 cells, where we knocked down NRF2 and further co-treated them with BTZ and nelfinavir (Figure S13). The mRNA levels of the inspected proteasome genes significantly decreased when NRF2 was downregulated compared to BTZ and NFV co-treated cells, but similar decrease was also observed with mock siRNA. Nevertheless, there is a decreasing tendency of the proteasome subunits mRNA levels, when comparing NRF2siRNA+BTZ+NFV and mocksiRNA+BTZ+NFV. This phenomenon will be inspected in the future.

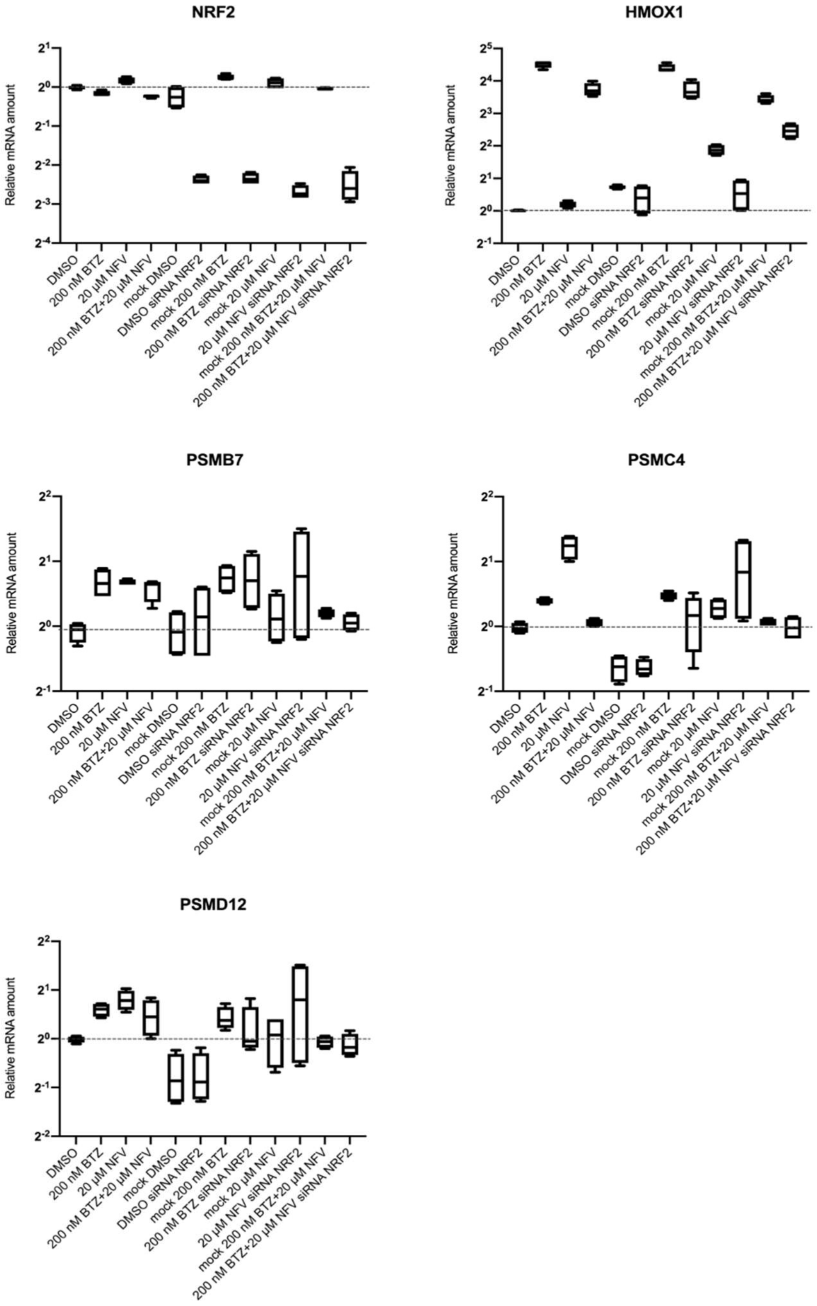

Figure S13. Quantitative RT-PCR analyses of the indicated genes are shown. NRF2 was downregulated in HEK293 cells by transfecting siRNA targeting NRF2 (sc-37030, Santa Cruz Biotechnology) using RNAiMAX (Invitrogen) and after 24h the cells were co-treated with 200 nM BTZ, and 20 µM NFV, for 16 h. Respective controls are indicated. RNA extracted from the cells was converted to cDNA and used for RT-qPCR with the primers listed in Table S1. mRNA levels of *GAPDH* were used for normalization. The boxes indicate interquartile ranges, while whiskers denote minimal and maximal values ($n = 4$).

Table S1. RT-qPCR primers used in the study.

Primer	Sequence (5′-3′)
DDI2-F	CTCCGAGGTGACCTTTTCCC
DDI2-R	CTGTGAGAGGTCTTTCCGCA
GAPDH-F	AATCCCATCACCATCTTCCA
GAPDH-R	TGGACTCCACGACGTACTCA
HMOX-1-F	ATGACACCAAGGACCAGAGC
HMOX-1-R	GTGTAAGGACCCATCGGAGA
NFE2L1-F	GCCCTGTTTCACTTATAGGGTCTAGA
NFE2L1-R	GGCAAAGAGAACATTTAGCAGCTT
NFE2L2-F	AGCGACGGAAAGAGTATGA
NFE2L2-R	TGGGCAACCTGGGAGTAG
POMP-F	GTGCAGCAGGTTCAGCGTCT
POMP-R	TGTGGCTCTCCCATGACTTCGC
PSMA7-F	CTGTGCTTTGGATGACAACG
PSMA7-R	CGATGTAGCGGGTGATGTACT
PSMB7-F	TGCAAAGAGGGGATACAAGC
PSMB7-R	GCAACAACCATCCCTTCAGT
PSMC4-F	GGAAGACCATGTTGGCAAAG
PSMC4-R	AAGATGATGGCAGGTGCATT
PSMD12-F	GTGCGCGACTGACTAAAACA
PSMD12-R	TAGGCAGAGCCTCATTTGCT

© 2020 by the authors. Licensee MDPI, Basel, Switzerland. This article is an open access article distributed under the terms and conditions of the Creative Commons Attribution (CC BY) license (http://creativecommons.org/licenses/by/4.0/).
